# Looking Forward

**Published:** 1890-02

**Authors:** S. H. Preston


### LOOKING FORWARD.

BY S. H. PRESTON.

No. 6.

During the last thirty-five years mechanical invention has wrought wonders in the industrial world. Steam and steel have supplanted human sinew, and machinery now mainly supplies the place of muscle in the former domains of drudgery. Especially is this so of the factory, which as a factor of wealth is rapidly forestalling the farm. But even the clod hopper is giving way to a gloved gentleman that tills his grounds in a gig, doing in a day a whole week's work of old fogy farmers.

The treadmills of toil are now turned by the tireless Titans of the elements, ironed to their tasks by Yankee ingenuity.

When a machine is made which does the work of a dozen men, it must follow that there will be at least eleven less men wanted in the line of labor to which it is applied. Every labor-saving contrivance serves to decrease the demand for manual labor. The more the different departments of industry are invaded by invention, the more that sinew succumbs to steel, the more men will there be in want of work. The inventions of Howe and Singer revolutionized some of the industries of domestic life, and rendered needless half the hands of needy needle-women. The automatic artisans of Lynn, the restless looms of Lowell, have crowded out the single-handed craftsmen and filled New England thoroughfares with tramps. In Massachusetts, 65,000 operatives care for 5,000,000 spindles and 100,000 looms. These figures furnish the clue to cheapening labor.

Flesh and blood cannot compete with the metallic mammoths whose food is fuel, whose nerves are steel and whose breath is steam. Not only do looms and iron arms labor for less than living men, but they do a hundred times the work and never flag from lack of food. Not only does machinery multiply the means of work, but it diversifies the kinds of work and leaves but little for hand-skilled labor. The result is lowering wages and lessening work. The prodigious progress in the processes of labor has so cheapened handiwork that hosts of the industrial classes are now of no service to themselves or society. Until there is a readjustment of muscle to machinery under the changed condition of things, manual toil will be a drug in the market, and there will be more workers than the world wants.

Every wheel whirled by water, every spindle stirred by steam, starts a tear to the sunken cheek of starving toil, and a sigh from the hopeless hearts of beggared bread-winners.

Labor, like every other marketable commodity, is regulated by the law of demand and supply. But it differs from other commodities in this, that it is not called into existence till it is bought. It cannot be kept in stock like store goods, and held back for a better market. The labor that is not sold to-day is not left over for to-morrow ; it is lost absolutely. A large portion of work people are forced to toil to-day for what will feed them to-morrow. Deprived of work to-day they wil be destitute to-morrow. And every additional idle day will increase their distress. But it is not so with the capitalist who buys labor. He can hold his commodity in reserve without loss, and often to advantage. He can put it at interest so it will augment from day to day, increase Sundays and while he sleeps—so that it will feed upon itself, so to speak. While the laborer is obliged to work for what wages he can get, and maybe is glad to get any, however meagre, the capitalist need not buy unless it be to his benefit. This makes him master of the labor market. So that in every battle between the two the latter is sure to win.

Of course labor is thus cheapened, because controlled by capitalists who will naturally pay as little as possible for it. By means of improved and newly invented machinery they are enabled to monopolize it more and more. Machines which make one man equal to twenty are mighty means for increasing the capital of manufacturers, but they deprive nineteen laborers out of every twenty of their employment, and multiply want among the working masses.

The cause of low wages and want in over-crowded communities is simply that the supply of willing workers exceeds the demand for work. In England and Continental Europe the clamorous idle laborers do not want charitable relief. They refuse to receive it. But they want government to give them work. Now government cannot alter the natural law of supply and demand as applied to labor. No government can force industry beyond its trade limit to any lasting advantage. It can clear impediments from commercial channels, stimulate trade, but cannot augment its amount. To employ an idle population in surplus production would be but to temporarily allay trouble without any permanent utility.

The amount of work will always depend upon the amount of capital that can be advantageously expended in wages; and whether the wages be high or low will depend upon the number of men in want of work. Suppose the entire capital of a country is applied to the payment of laborers. Its income, portioned into weekly wages, would suffice for paying a certain sum to a certain number of workers. At first the amount is ample to provide all with the comforts of life. The workers increase their number according to the law of population, while their is no corresponding increase of capital. Soon the wages will scarcely suffice for the necessaries of life, and erelong they will not be enough to buy bread for all. Then some of the desperate destitute resort to riotous proceedings, and compel the capitalists to advance wages. What will be the inevitable result? Simply that the increased wages can be paid to but a part of the hands, and the rest will receive none. The higher the wages are raised the fewer there will be to share them and the more who will be left without any. It is merely a matter of necessities of demand and supply. And the rate of wages will ever be regulated by the amount of capital, the amount of work wanted, and the number of hands to do that work. So that there is no way of raising wages except by increasing capital, which increases the demand, or by reducing the hands, which reduces the supply.

Such is the necessary relation between labor and capital under equitable conditions of compensation, or where the wages are properly proportioned to the work performed.

But it is a fact that in our fluctuating and inequitable state of affairs, some do not receive enough remuneration and others too much. The average of wages in the United States is $450 per annum. And in order to pay some people, as the President, $50,000 a year, other people must be paid pretty low. Wages are better equalized in our country than in any other, but it is a pernicious principle which permits the president of a foundry company to pocket ten times the pay of a puddler. Here where the laborer has so large a liberty and holds a ballot, where a rail-splitter, a tanner, or canal boatman may be raised to the highest rank in the Republic, capital can never attain a dangerous attitude. That there are more working people than can be profitably provided with employment is reason rather for regret than deprecation.

All of advanced age remember when the families of day laborers were in a comparatively comfortable condition—when work was plenty and good wages were promptly paid. That is, good wages according to the cost of living then. Though they were then twenty or twenty-five per cent. of what they are at present, all the commodities for the comfort of life were three hundred per cent. less.

Now there is something wrong. There are mendicants and millionaires. A few generations ago there were no millionaires. They are now starting up like mushrooms. It is less than half a century since San Francisco was settled, and it now numbers over one hundred millionaires. A man falls over in a fit on Fifth avenue, New York, leaving $200,000,000 to his heirs. Another who commenced his commercial career with a mouse trap is now worth $100,000,000. In the boxes of the Metropolitan Opera House at an evening's entertainment were seated the possessors of $500,000,000. And this in the city where a man once chalked on the walls of his cell in the Tombs, "In New York the spires of 342 churches, worth $41,120,000, point heavenward. I am here for stealing a loaf of bread for my starving child." And children do even starve in this charitable city in which one church corporation alone, Trinity, holds nearly $100,000,000 of wealth, where one cathedral is already erected at a cost of millions and another is planned upon an estimate of $8,000,000. The billionaire will be here by and by, but beggars are multiplying.

Well, here are heaps of capital, here are hundreds of millionaires, and here also are hosts of honest, hungry wage-workers and paupers enough to populate a State. Superfluity on one side and starvation on the other. What does it mean? It means the domination of mammon. That capital is being cornered by cupidity. That the gold that is enough for the many is being gobbled up into the money bags of the few. But why do the many who, in addition to their advantage of numbers, enjoy the right to better their condition by the ballot, suffer the fruits of their labor to be filched from them by the few? Why do they let themselves be cheated out of the capital they have created? This is the strangest feature of this strange state of affairs.

Here in this great representative Republic the ballot levels a Vanderbilt with the veriest beggar. And if working-men, being in the majority, cannot command better wages or secure what they earn, they have no cause for complaint. We would have little pity for a party of missionaries who would permit themselves to be eaten by a smaller company of cannibals. A robust man with a bull-dog pistol who allows himself to be robbed by a beardless boy, would do better not to whine at his loss.

It is true we are in an unsettled, a still selfish and see-saw state of society. Evils and inequalities prevail in all the relations of life. But if things are righted it must be by the majority, the laborers with the ballot, instead of dreaming of Bellamy's ideal Boston, or founding Familisteres and Topolobampos in foreign lands.

To summarize the matter: Theworking masses have multiplied faster than the means for their maintenance. Machinery has multiplied manufactures beyond the market demand. A stupendous surplus stock of all sorts has been produced, crippling capital and choking the channels of commerce.

To illustrate: During the next twenty-five years, there will be a demand for just a certain number of palace cars. These may be made in a short time at the town of Pullman, Ill.; then the makers of them must remain idle or seek some other line of labor, unless more capital is converted into cars to be stored away for the next quarter of a century, when, according to the past rate of increase, the laboring populace will have doubled their numbers. Then there will be twice as many car-builders as now, with no demand for their work. Thus it is at all the great manufacturing centers of the country.

Again, new mechanic forces and contrivances are being brought into use which do away with manual toil, and by which large numbers of laborers are dispensed with or displaced. When electric motor street cars come into general use the occupation of thousands of horse drivers will be gone. And all the while the vast army of the idle is augmented by the foreign influx. There is destitution among Dakota farmers, while great and greedy and conscienceless corporations are conspiring to control the capital of the country. And all these things are pressing upon us that perplexing population problem.

